# Effects of transgenic silk materials that incorporate FGF‐7 protein microcrystals on the proliferation and differentiation of human keratinocytes

**DOI:** 10.1096/fba.2020-00078

**Published:** 2020-10-20

**Authors:** Rina Maruta, Keiko Takaki, Yuka Yamaji, Hideki Sezutsu, Hajime Mori, Eiji Kotani

**Affiliations:** ^1^ Department of Applied Biology Kyoto Institute of Technology Kyoto Japan; ^2^ Institute of Agrobiological Sciences National Agriculture and Food Research Organization Tsukuba Ibaraki Japan

**Keywords:** 3D epidermal model, FGF‐7, polyhedral, transgenic silkworm

## Abstract

The silk glands of silkworms produce large quantities of fibroin, which is a protein that can be physically processed and used as a biodegradable carrier for cell growth factors in tissue engineering applications. Meanwhile, protein microcrystals known as polyhedra, which are derived from cypovirus 1, have been used as a vehicle to protect and release encapsulated cell growth factors. We report the generation of transgenic silkworms that express recombinant fibroblast growth factor‐7 (FGF‐7) fused with the polyhedron‐encapsulating signal in polyhedra produced in the middle (MSG) and posterior (PSG) silk glands. Immunofluorescence showed that polyhedra from silk glands are associated with FGF‐7. The MSG and PSG from transgenic silkworms were processed into fine powdery materials, from which FGF‐7 activity was released to stimulate the proliferation of human keratinocyte epidermal cells. Powders from PSGs exhibited higher FGF‐7 activity than those from MSGs. Moreover, PSG powder showed a gradual release of FGF‐7 activity over a long period and induced keratinocyte proliferation and differentiation in 3D culture to promote the formation of stratified epidermis expressing positive differentiation marker proteins. Our results indicate that powdery materials incorporating the FGF‐7‐polyhedra microcrystals from silk glands are valuable for developing cell/tissue engineering applications in vivo and in vitro.

**Abbreviations**
3D: three‐dimensional
CPV: cypovirus 1
DsRed: discosoma sp. Red
EGFP: enhanced green fluorescent protein
ELISA: enzyme‐linked immunosorbent assay
FGF‐2: fibroblast growth factor‐2
FGF‐7: fibroblast growth factor‐7
FH: fibroin heavy chain gene
H1: polyhedron‐encapsulation signal helix‐1
H1/FGF‐7: FGF‐7 fused with H1‐tag
H1/FGF‐7‐polyhedra: H1/FGF‐7‐encapsulated polyhedra
ITR: inverted terminal repeat
KCM: keratinocyte‐conditioned medium
KMO: kynurenine 3‐monooxygenase
MMP: matrix metalloproteinase
MSG: middle silk gland
MSGP: middle silk gland powder
NHEKs: normal human epidermal keratinocytes
PSG: posterior silk gland
PSGP: posterior silk gland powder
rhFGF‐7: recombinant human FGF‐7
S1: Sericin 1 gene
Sf21 cells: *Spodoptera frugiperda* IPLB‐SF21‐AE cells
SGP: silk gland powder
UAS: upstream activation sequence

## 1 INTRODUCTION

The middle silk gland (MSG) and posterior silk gland (PSG) of silkworms, *Bombyx mori*, produce large quantities of silk thread proteins.^1^ Recombinant versions of proteins produced in these MSGs^1^, ^2^, ^3^, ^4^ and PSGs^5^, ^6^, ^7^, ^8^ are used in protein engineering. The PSG expresses the biopolymer protein fibroin, which is highly biodegradable, biocompatible, and shows low immunostimulatory activity, making it suitable to produce surgical sutures^5^ and grafts for revascularization,^9^ bone tissue regeneration,^10^ and cutaneous wound healing.^11^, ^12^

Posterior silk glands engineered to express cell growth factors could be processed into biomedical materials, such as powders with high concentrations of fibroin, which could control the proliferation of cells in vivo and in vitro. However, one potential disadvantage of this system is that cell growth factors, including the human fibroblast growth factor‐7 (FGF‐7), are typically unstable under harsh environmental conditions, causing them to have a short half‐life.^13^, ^14^

The *B*.*mori* cypovirus 1 (CPV), which is a member of the family *Reoviridae*, produces proteinaceous occlusion bodies known as polyhedra that include microcrystals of the protein polyhedrin, which has attracted attention for its potential ability to encapsulate and protect proteins.^15^, ^16^, ^17^, ^18^ This virus infects cells in the silkworm digestive tract, producing large numbers of polyhedra. Progeny viruses are occluded in polyhedra that protect against infectivity over the long term and in the outdoor environment.^15^ We previously showed that polyhedra could encapsulate diverse foreign proteins, such as fluorescence proteins,^16^, ^17^, ^18^, ^19^ cytokines,^20^, ^21^, ^22^, ^23^ and fusion proteins comprising enzymes^24^ with an N‐terminal polyhedron‐encapsulation signal alpha‐helix sequence H1 and C‐terminal VP3‐tag that are expressed during polyhedron crystallization in cultured insect cells. In addition, the cytokine activities in polyhedra are stable in the long term.^16^ The remarkable stability of polyhedra‐encapsulated proteins suggests that these systems could be robust as sustained‐release carriers of cytokines and other proteins for tissue engineering.^16^, ^20^, ^21^, ^22^, ^23^, ^24^

We also recently reported that the introduction of polyhedra into an in vitro neurodifferentiation cell culture model induced the release of active neurotrophin from polyhedra upon gradual polyhedra proteolysis mediated by small amounts of cell‐derived matrix metalloproteinases.^23^ These findings highlight the potential biomedical applications of polyhedra that encapsulate cytokines, such as FGF‐7, inside silkworm silk glands. These silk glands can be processed to yield silk materials incorporating cytokine‐polyhedra to control cell proliferation.

Our earlier studies showed that protein expression systems involving bioengineered silk glands could effectively produce active cytokines, such as fibroblast growth factor‐2 (FGF‐2; ref. 7). Here, we focused on the generation of transgenic silkworms using PSGs and MSGs that produce polyhedron‐encapsulated FGF‐7. FGF‐7 is used for establishment of a three‐dimensional (3D) culture system that serves as an in vitro model epidermis.^25^ We examined whether the biological activity of FGF‐7 can be released from the polyhedra to induce keratinocyte proliferation and epidermal differentiation of cells in supplement‐free culture. We also investigated the effectiveness of FGF‐7 activity released from polyhedra that are incorporated in PSGs in 3D keratinocyte cultures to inform the construction of a human epidermis model.

## 2 MATERIALS AND METHODS

### 2.1 Silkworms

Nondiapausing *w1*‐*pnd* lines that lay nonpigmented eggs were used to generate transgenic silkworms.^26^ Silkworm larvae were aseptically reared at 25°C on an artificial diet (Aseptic Sericulture System Laboratory).

### 2.2 Cultured cells

Normal human epidermal keratinocytes (NHEKs; Kurabo, Osaka, Japan) were cultured using Humedia KG‐2 medium (Kurabo) supplemented with insulin (10 µg/ml), human epidermal growth factor (EGF) (0.1 ng/ml), hydrocortisone (0.67 µg/ml), gentamycin (50 µg/ml), amphotericin (50 ng/ml), and bovine pituitary extract (0.4% V/V) in 5% CO_2_ at 37°C. Keratinocytes in the third passage were cultured using a defined keratinocyte‐serum free medium (DK‐SFM) supplemented with defined keratinocyte‐SFM growth supplement that included insulin, EGF, and FGF (Thermo Fisher Scientific).

### 2.3 Plasmid construction and microinjection

A FGF‐7 sequence fused with a polyhedron‐encapsulation signal helix‐1, H1 (H1/FGF‐7) and *Nhe*I sites at both ends was amplified using polymerase chain reaction with the primers 5′‐AAAGCTAGCATGGCAGACGAGCAG‐3′ (forward) and 5′‐AAAGCTAGCTTAAGTTATTGCCTAGGAAG‐3′ (reverse) and a vector previously used for the construction of recombinant FGF‐7‐expressing baculovirus as the template to construct donor plasmids for transgenesis.^16^ The amplified fragments were digested with *Nhe*I and inserted into the *Bln*I site downstream of the upstream activation sequence (UAS) in the pBacMCS[UAS/SV40,3×P3‐enhanced green fluorescent protein (EGFP)] plasmid.^27^ The resulting plasmid, pBacMCS[UAS‐H1FGF‐7/SV40,3×P3‐EGFP], was purified from the bacterial culture using QIAGEN Midi (Qiagen).

To generate transgenic silkworms that expressed H1/FGF‐7 in their silk glands, we performed microinjection as previously described.^28^ We observed EGFP present in the eyes in G1 eggs using fluorescence microscopy with an Olympus IX71 light microscope (Olympus). The transgenic line carrying the gene for H1/FGF‐7 (UAS‐H1/FGF‐7 line) was established through repeated sibling mating of selected individuals exhibiting EGFP expression in the eyes.

Genomic DNA was isolated from the UAS‐H1/FGF‐7 line, and the sequences at the borders of the transgene insertion were assessed using inverse PCR, and database analysis was conducted using the KAIKOBLAST database (http://kaikoblast.dna.affrc.go.jp). DNA was digested with *Sau*3A1 (Takara Bio Inc.) and circulated using Ligation High ver.2 (Takara Bio Inc.). The ligated DNA was amplified using KOD plus version 2 DNA polymerase (Toyobo) under standard conditions with primers designed based on the left‐ and right‐hand region of the vector (left primer pair, 5′‐GCGTGAGTCAAAATGACGCATGAT‐3′ and 5′‐ATCAGTGACACTTACCGCATTGACA‐3′ and right primer pair, 5′‐CCTCGATATACAGACCGATAAAACAC‐3′ and 5′‐AACTTTTATGGCGCGCCATCGAAT‐3′). The purified fragments were sequenced directly (ABI PRISM^Ⓡ^ 310 Genetic Analyzer, Applied Biosystems, Foster City, CA, USA) using primers for the left (5′‐GACTGAGATGTCCTAAATGCACAG‐3′) and right boundary (5′‐GACCGATAAACACATGCG‐3′) of the vector.

### 2.4 Generation of transgenic silkworms that express H1/FGF‐7 and polyhedra

**FIGURE 1:**
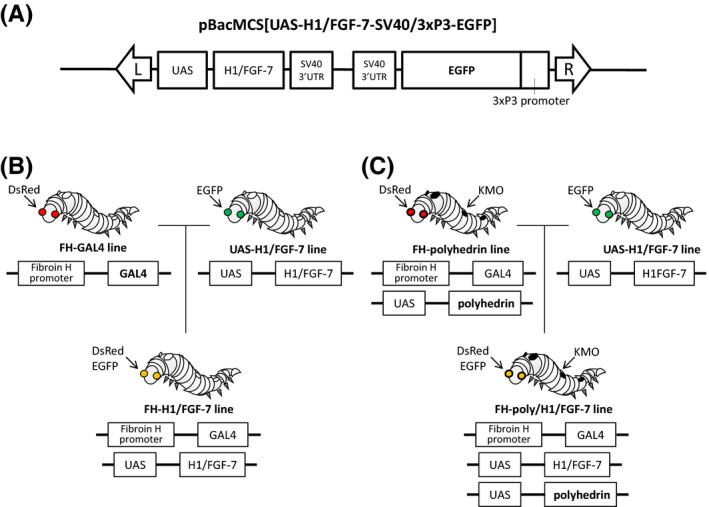
Generation of transgenic silkworms that express H1/FGF‐7 in the posterior silk gland. *A*) Schematic representation of the plasmid vector used to generate transgenic silkworms. The plasmid vector pBacMCS[UAS‐H1/FGF‐7‐SV40/3xP3‐EGFP] encoding H1/FGF‐7 was used to generate the transgenic line termed UAS‐H1/FGF‐7. The *piggyBac* right and left inverted terminal repeats (ITRs) (L and R) are indicated by arrows. *B*) Generation of the FH‐H1/FGF‐7 line. The FH‐GAL4 line^29^ was mated with the UAS‐H1/FGF‐7 line to generate the FH‐H1/FGF‐7 line in which expression of EGFP and discosome sp. Red (DsRed) as genetic markers is visible in the eyes. *C*) Generation of the FH‐poly/H1/FGF‐7 line. The FH‐polyhedrin line was mated with the UAS‐H1/FGF‐7 line to generate the FH‐poly/H1/FGF‐7 line with the expression of EGFP and DsRed visible in the eyes and kynurenine 3‐monooxygenase (KMO) specific expression in the skin,^7^ as genetic markers.

The UAS‐H1/FGF‐7 line was mated with PSG‐ (FH‐GAL4; ref. 29) or MSG‐specific (S1‐GAL4; ref. 30) Gal4 driver lines to obtain FH‐H1/FGF‐7 (Figure 1B) or S1‐H1/FGF‐7 lines (Figure S1A) expressing H1/FGF‐7 under the control of the fibroin heavy chain gene (*FH*) promoter in PSGs or the Sericin 1 gene (*SI*) promoter in MSGs. Both promoters are active during the larval spinning stage.^29^, ^30^ The transgenic line carrying CPV polyhedrin (UAS‐polyhedrin line; ref. 7) was then mated with the FH‐GAL4 or S1‐GAL4 line to obtain an FH‐polyhedrin line expressing polyhedrin in PSGs (Figure 1C) or S1‐polyhedrin in MSGs (Figure S1B). Finally, the UAS‐H1/FGF‐7 line was mated with the FH‐polyhedrin line to obtain the FH‐poly/H1/FGF‐7 line (Figure 1C), which expresses both H1/FGF‐7 and polyhedrin in PSGs under the control of the *FH* promoter. The S1‐polyhedrin and UAS‐H1/FGF‐7 lines were mated in the same way to obtain the S1‐poly/H1/FGF‐7 line (Figure S1B), which expressed both H1/FGF‐7 and polyhedrin in MSGs under the control of the *S1* promoter.

### 2.5 H1/FGF‐7 immunoblotting

H1/FGF‐7 was purified from the PSG of larvae of the FH‐H1/FGF‐7 line at the spinning stage by immunoprecipitation using a Dynabeads Protein A kit (Thermo Fisher Scientific) following the manufacturer's instructions. A measure of 50 mg of PSGs from FH‐H1FGF‐7 or *w1*‐*pnd* control larvae were dissolved in RIPA buffer (Nacalai Tesque) using sonication. After centrifugation at 6,000 × *g* for 5 min, the supernatants were incubated with protein A Dynabeads bound to an anti‐FGF‐7 antibody (ReliaTech GmbH, Wolfenbüttel, Germany) overnight at 4°C. Dynabead‐Ab‐Ag complexes were washed with the washing buffer supplied in the kit and then lysed in a sample buffer for immunoblotting. Fifty‐thousand cubes of empty polyhedra with no H1/FGF‐7 and H1/FGF‐7‐encapsulated polyhedra^16^ produced in baculovirus‐infected Sf21 cell lines were used as negative and positive controls, respectively. Proteins in the samples were separated by 12.5% SDS‐PAGE, transferred onto PVDF membranes (GE Healthcare Bioscience) and blocked with Blocking One (Nacalai Tesque). After blocking, membranes were incubated in a primary antibody solution containing 1:5,000 anti‐FGF‐7 antibody, washed in PBS (−), and incubated in a secondary antibody solution containing 1:5,000 goat anti‐rabbit IgG conjugated with horseradish peroxidase (Bio‐Rad). Target protein bands were visualized after incubation with detection reagent (GE Healthcare Bioscience).

The MSGs from S1‐H1/FGF‐7 larvae were examined in the same way using the abovementioned immunoblotting procedure.

### 2.6 Immunofluorescence of polyhedra from posterior or middle silk glands

Anti‐Human FGF‐7 was directly labeled with HiLyter Fluor^TM^ 555 following the manufacturer's instructions (HiLyter Fluor^TM^ 555 Labeling Kit‐NH_2_, Dojindo Laboratories). Polyhedra from silk gland powders (SGPs) suspended in PBS (–) were collected using sonication and centrifugation. Polyhedra of PSGs from FH‐poly/H1/FGF‐7 or FH‐polyhedrin larvae at the spinning stage were placed on the bottom of glass‐based dishes (Iwaki glass). After air drying, polyhedra were blocked with Blocking One solution and incubated in a primary antibody solution containing anti‐polyhedrin antibody diluted 1:1,000 and then washed in PBS (–) at room temperature for 15 min. After washing, polyhedra were further incubated in a secondary antibody solution containing 1:500 Alexa Fluor 488‐conjugated anti‐rabbit IgG (Invitrogen). Then, they were washed in PBS (–). Next, the polyhedra were incubated in an antibody solution containing HiLyter Fluor^TM^ 555‐labeled anti‐FGF‐7 antibody diluted 1:500, washed in PBS (−), and examined using an Olympus Fluoview FV1000‐IX81 confocal microscope (Olympus) with a 100x objective lens. The MSG polyhedra from S1‐poly/H1/FGF‐7 or S1‐polyhedrin larvae were examined with an anti‐FGF‐7 antibody, as described above.

### 2.7 Enzyme‐linked immunosorbent assay analysis of H1/FGF‐7 released from silk gland materials

The amount of H1/FGF‐7 released from FH‐poly/H1/FGF‐7 larvae PSGs was determined using an enzyme‐linked immunosorbent assay (ELISA) using an anti‐FGF‐7 antibody. The PSGs from spinning larvae of *w1*‐*pnd* and FH‐poly/H1/FGF‐7 were freeze‐dried and crushed into fine powders, which were called posterior silk gland powders (PSGPs). Empty polyhedra and H1/FGF‐7‐encapsulated polyhedra (H1/FGF‐7‐polyhedra) were prepared using similar methods to those described by Ijiri et al.^1^ in the recombinant baculovirus‐infected *Spodoptera frugiperda* cells (Sf21 cells). Keratinocyte‐conditioned medium (KCM) was harvested from NHEKs cultured (3.0 × 10^4^ cells /ml) in DK‐SFM without a growth supplement for 48 h. Over 14 days, 5.0 × 10^5^ cubes of polyhedra and 1 mg of PSGPs were added to KCM using a cell culture insert filter. Media were collected on days 3, 7, 10, and 14, and the quantity of FGF‐7 in the collected culture medium was determined using a Human KGF/FGF‐7 Quantikine ELISA kit (R&D Systems), following the manufacturer's instructions. The amount of soluble H1/FGF‐7 was quantified by measuring the absorbance at 450 nm with a microplate reader (Bio‐Rad Model 680, Bio‐Rad). Media incubated with empty polyhedra or PSGPs prepared from *w1*‐*pnd* larvae were used as blanks for the ELISA analysis for the media incubated with H1/FGF‐7‐polyhedra or PSGP from FH‐poly/H1/FGF‐7 larvae, respectively.

### 2.8 Detection of phosphorylation of p44/p42 mitogen‐activated protein kinase

The PSGPs from FH‐polyhedrin, FH‐H1/FGF‐7, and FH‐poly/H1/FGF‐7 spinning larvae and middle silk gland powders (MSGPs) from S1‐polyhedrin, S1‐H1/FGF‐7, and S1‐poly/H1/FGF‐7 spinning larvae were obtained as described above. Then, these PSGPs and MSGPs were stored at −80°C until use.

NHEKs were seeded onto six‐well tissue culture plates (Iwaki glass) at 2.5 × 10^4^ cells/well and cultured to confluence in Humedia KG‐2 media for 4 days. After incubation, the medium was replaced with Humedia KB‐2 medium (Kurabo) lacking growth supplement and incubated for 16 h to induce starvation. After starvation, a cell culture insert (Corning) was placed in the well, and 10 mg of PSGPs or MSGPs from a series of silkworms were suspended in Humedia KB‐2 and added onto the filter of each cell culture insert. Recombinant human FGF‐7 (rhFGF‐7; 100 ng/ml; Wako Chemical) was added as a positive control. Cells were incubated with PSGPs or MSGPs for 1 h, washed with HEPES buffer (Kurabo), and dissolved in 100 µl SDS‐PAGE sample buffer (50 mM Tris‐HCl, pH 6.8, 100 mM dithiothreitol, 2.0% SDS, 0.1% bromophenol blue, 10% glycerol). Each cell lysate was analyzed using immunoblotting as described above, with Phospho‐p44/p42 MAP Kinase (Thr202/Tyr204) antibody (1:2,500; Cell Signaling Technology, Danvers, MA, USA) or p44/p42 MAP Kinase antibody (1:5,000; Cell Signaling Technology) as the primary antibody. Protein bands were detected using an HRP‐conjugated anti‐rabbit IgG (1:5,000, Bio‐Rad).

### 2.9 Assay for keratinocyte proliferation

To measure NHEK proliferation, we used a Cell Counting Kit‐8 (CCK‐8, Dojindo Laboratories), which detects intracellular dehydrogenase using water‐soluble tetrazolium salt WST‐8. NHEKs were seeded at 5 × 10^3^ cells/well onto 24‐well culture plates (Iwaki glass) in DK‐SFM without growth supplement. Cell culture inserts were placed in the well, and suspensions of PSGPs (50, 100, or 200 µg) or MSGPs (50, 250, or 500 µg) in DK‐SFM were added onto the filter of each cell culture insert. rhFGF‐7 (2, 4, or 10 ng/ml) was used as a positive control. After culturing for 72 h at 37°C in 5% CO_2_, 10% (v/v) CCK‐8 was added to the medium and incubated for 2 h at 37°C under 5% CO_2_. The absorbance of media was then measured at 450 nm using a microplate reader.

## 3 3D culture of keratinocytes

For 3D cultures, 800 µg PSGPs from FH‐polyhedrin or FH‐poly/H1/FGF‐7 larvae was mixed in 250 µL of collagen solution (Cellmatrix Type I‐A; Nitta Gelatin Inc). The gelated collagen was poured into a RING‐12 cloning ring (φ10 mm; Iwaki glass) in a six‐well plate and incubated for 1 h at 37°C in 5% CO_2_. In this experiment, DK‐SFM was used as a basal medium without growth supplement, and basal medium mixed with 1.2 mM Ca^2+^ was used as a differentiation medium. A measure of 4 mL of basal medium was added to each well, and then 2.0 × 10^5^ NHEKs suspended in 200 µL of basal medium were deposited on a collagen gel. After the NHEKs were submerged in the basal media for 2 days, the cloning rings were removed, and the basal medium was discarded. Then, 1 ml of the differentiation medium was added into each composite culture, and it was raised to the air‐liquid interface for 14 days. Each differentiation medium was replaced twice weekly. 3D cultures of keratinocytes on the gel were also incubated with basal and differentiation media containing 100 ng/mL rhFGF‐7.

After 14 days, the 3D keratinocyte cultures were fixed in 4% formaldehyde overnight. The fixed 3D cultures were then embedded in optimal cutting temperature compound (Sakura Finetek Japan, Tokyo, Japan) and cut into 20 micron‐thick sections, which were placed on glass slides. The sections were hydrated and stained with hematoxylin and eosin (HE) before observing them with a light microscope (Olympus IX71, Olympus) with a 40× objective lens. For immunohistochemistry, sections were incubated with primary antibodies for keratin 14 (Invitrogen), keratin 10 (Abcam), loricrin (BioLegend), and filaggrin (Santa Cruz Biotechnology). After washing in PBS(−), sections were incubated with Alexa Fluor 488‐conjugated anti‐rabbit IgG (Invitrogen) or Alexa Fluor 594‐conjugated anti‐mouse IgG (Invitrogen). Then, the nuclei were stained with 4′,6‐diamidino‐2‐phenylindole. Immunofluorescence imaging of the sections was conducted using confocal microscopy with a 40× objective lens.

### 3.1 Data analysis

All results are expressed as the mean ±standard deviation (SD) of triplicate assays (N = 3 independent samples). Data were analyzed using a one‐way analysis of variance followed by Tukey's post hoc test for pair‐wise comparisons. Differences were considered to be significant at *p* < 0.05 and *p* < 0.01.

## 4 RESULTS

### 4.1 Establishment of transgenic silkworms expressing H1/FGF‐7 in posterior silk glands

**FIGURE 2:**
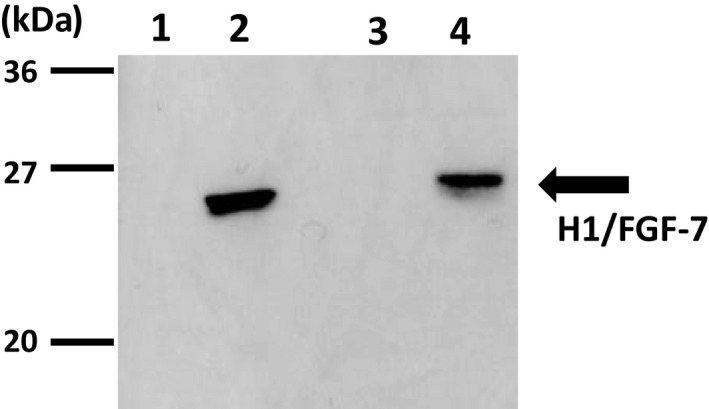
Immunoblotting analysis of H1/FGF‐7 expressed in posterior silk glands. Protein samples from 50,000 empty polyhedra cubes (negative control; lane 1) and H1/FGF‐7‐polyhedra (positive control; lane 2) produced in baculovirus‐infected Sf21 cells, in addition to proteins immunoprecipitated from 50 mg PSGs from *w1*‐*pnd* (lane 3) and FH‐H1/FGF‐7 larvae (lane 4) were electrophoresed on 12.5% SDS‐PAGE and analyzed by immunoblotting with anti‐FGF‐7 antibodies. Protein size markers are indicated on the left

We first analyzed the expression of FGF‐7 with an N‐terminal polyhedron‐encapsulation signal H1 (H1/FGF‐7; ref. 16) in the silk glands of transgenic silkworms. A donor plasmid (Figure 1A) was used for transgenesis of the silkworms and to establish a UAS‐H1/FGF‐7 line carrying the transgene. The presence of the transgene in chromosome 8 of the UAS‐H1/FGF‐7 line was confirmed using inverse PCR (Table S1). The FH‐H1/FGF‐7 line was generated by mating the UAS‐H1/FGF‐7 line with an FH‐GAL4 line^29^ carrying the GAL4 gene under the control of the *FH* promoter (Figure 1B). Immunoprecipitation with an anti‐FGF‐7 antibody was performed to examine PSG samples from the FH‐H1/FGF‐7 line in the spinning stage (Figure 2). Neither the negative control sample of recombinant baculovirus‐produced empty CPV polyhedra in Sf21 cell lines (lane 1) nor immunoprecipitated proteins of PSGs in *w1*‐*pnd* larvae (lane 3) showed bands for H1/FGF‐7 in immunoprecipitation. In contrast, PSG proteins immunoprecipitated from FH‐H1/FGF‐7 larvae (lane 4) showed signals for H1/FGF‐7 expression (calculated molecular mass ~24 kDa) as did the positive control sample of recombinant, baculovirus‐produced polyhedra encapsulating H1/FGF‐7 as previously described (lane 2; ref. 16).

**FIGURE 3:**
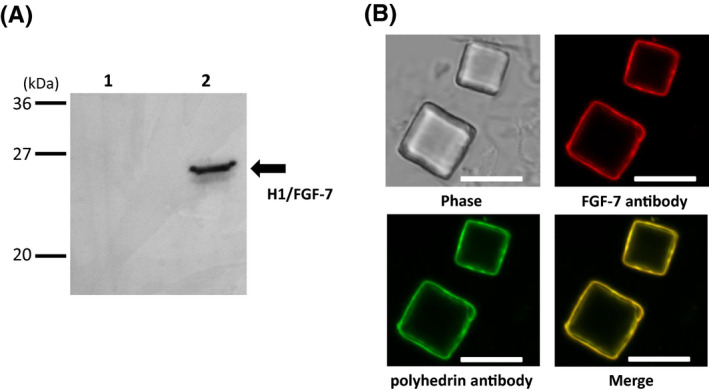
Detection of H1/FGF‐7 associated with polyhedra. A, Detection of H1/FGF‐7 in polyhedra using immunoblotting. Polyhedra (50,000 cubes) collected from PSGs of FH‐polyhedrin (lane 1, ref. 7) and FH‐poly/H1/FGF‐7 larvae (lane 2) were analyzed using immunoblotting with anti‐FGF‐7 antibodies. B, Confocal microscopy analysis of H1/FGF‐7 and polyhedrin expression on polyhedra by immunofluorescence. Polyhedra collected from PSGs of FH‐poly/H1/FGF‐7 larvae were fixed on a glass‐base dish and examined using immunofluorescence with anti‐FGF‐7 antibody and anti‐polyhedrin antibody. Scale bars, 10 µm

The FH‐poly/H1/FGF‐7 line was generated by mating the UAS‐H1/FGF‐7 line with the FH‐polyhedrin line that was previously shown to produce polyhedra in the PSG (Figure 1C; ref.7). Polyhedra (50,000 cubes) were collected from PSGs of FH‐poly/H1/FGF‐7 larvae at the spinning stage and analyzed using immunoblotting with an anti‐FGF‐7 antibody (Figure 3A). The same number of polyhedra in PSGs from FH‐polyhedrin larvae were analyzed in the same way as a negative control, and no H1/FGF‐7 was detected (Figure 3A, lane 1). Together, these results demonstrate that H1/FGF‐7 is present in polyhedra from FH‐poly/H1/FGF‐7 larvae (Figure 3A, lane 2).

Using immunofluorescence with anti‐FGF‐7 and anti‐polyhedrin antibodies to examine the association of H1/FGF‐7 with polyhedra in PSG collected from FH‐poly/H1/FGF‐7 larvae, we detected fluorescent anti‐FGF‐7 signals and anti‐polyhedrin signals on the surface of polyhedra from FH‐poly/H1/FGF‐7 line PSGs (Figure 3B). This result is consistent with that of Ijiri et al,^1^ who detected fluorescent proteins with N‐terminal H1 inside recombinant baculovirus‐produced polyhedral,^16^ and thereby demonstrated that H1‐fused foreign proteins were present inside of polyhedra due to H1 associating with the polyhedrin groove domain consisting of tyrosine cluster to tightly link the polyhedrins to each other during polyhedron crystallization. We only found an antibody signal at the surface (Figure 3B), even though H1/FGF‐7 was present inside the polyhedra. This is because the FGF‐7 antibody could not enter into the polyhedra. These results suggested that expressed H1/FGF‐7 was encapsulated into the polyhedra in the PSGs of FH‐poly/H1/FGF‐7 larvae.

The UAS‐H1/FGF‐7 line was mated with an S1‐GAL4 line to produce the S1‐H1/FGF‐7 line that expressed Gal4 under the control of the *S1* promoter to generate a transgenic silkworm expressing H1/FGF‐7 in MSGs (Figure S1A). We confirmed H1/FGF‐7 expression in MSGs from S1‐H1/FGF‐7 spinning larvae using immunoblotting with the anti‐FGF‐7 antibody (Figure S1C). The UAS‐H1/FGF‐7 line was then mated with the S1‐polyhedrin line to obtain transgenic S1‐poly/H1/FGF‐7 worms that express FGF‐7 and polyhedra in MSGs at the spinning stage (Figure S1B). Immunofluorescence analysis showed that an anti‐FGF‐7 signal could be detected on the surface of polyhedra collected from MSGs of S1‐poly/H1/FGF‐7 larvae, but not on polyhedra from S1‐polyhedrin larvae MSGs (Figure S1D). Restriction of H1/FGF‐7 fluorescence to the polyhedra surface was likely due to the inability of the antibody to enter the polyhedra. These results suggest that MSGs of S1‐poly/H1/FGF‐7 larvae produced polyhedra that encapsulated H1/FGF‐7.

### 4.2 Release of FGF‐7 from posterior silk gland powders incorporating FGF‐7‐polyhedra

**FIGURE 4:**
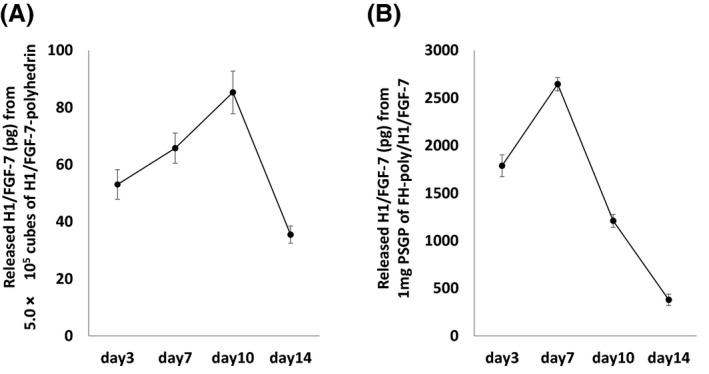
Release of H1/FGF‐7 from posterior silk gland powders (PSGPs) incorporating FGF‐7‐polyhedra. The amount of H1/FGF‐7 released from A, H1/FGF‐7‐polyhedra (5.0 × 10^5^ cubes) or B, Posterior silk gland powders (1.0 mg) from FH‐poly/H1/FGF‐7 larvae were examined using an enzyme‐linked immunosorbent assay (ELISA) with anti‐FGF‐7 antibody. Each sample was added into keratinocyte‐conditioned media using the filter of a cell culture insert (see MATERIALS AND METHODS) and incubated for 14 days. The medium was changed and collected on days 3, 7, 10, and 14. The amount of FGF‐7 in the collected culture medium was determined using an ELISA. Data shown are the means ±standard deviation (SD) of triplicate assays (N = 3 independent samples)

Next, we focused on the ability of H1/FGF‐7‐polyhedra in silk gland material to influence keratinocyte proliferation. We recently reported that upon introduction into cell culture, polyhedra are gradually proteolyzed by cell‐derived matrix metalloproteinases (MMPs) and release encapsulated proteins into the culture medium.^23^ The PSGs from FH‐poly/H1/FGF‐7 larvae were processed into a fine powder (PSGP; 1.0 mg) and added to the KCM that would contain keratinocyte MMPs to assess the biological properties of H1/FGF‐7‐polyhedra in SGPs. The PSGPs were incubated in culture for 14 days, and media were collected on days 3, 7, 10, and 14 to examine H1/FGF‐7 release by ELISA (Figure 4). We also measured the released H1/FGF‐7 from H1/FGF‐7‐polyhedra (5.0 × 10^5^ cubes) produced in baculovirus‐infected Sf21 cells. Five times the amount of polyhedra required for inducing keratinocyte proliferation was used.^16^ Until day 14, H1/FGF‐7 was continuously detected in media incubated with H1/FGF‐7‐polyhedra (Figure 4A) and PSGPs of FH‐poly/H1/FGF‐7 (Figure 4B).

### 4.3 Biological activity of H1/FGF‐7 expressed in silk glands

**FIGURE 5:**
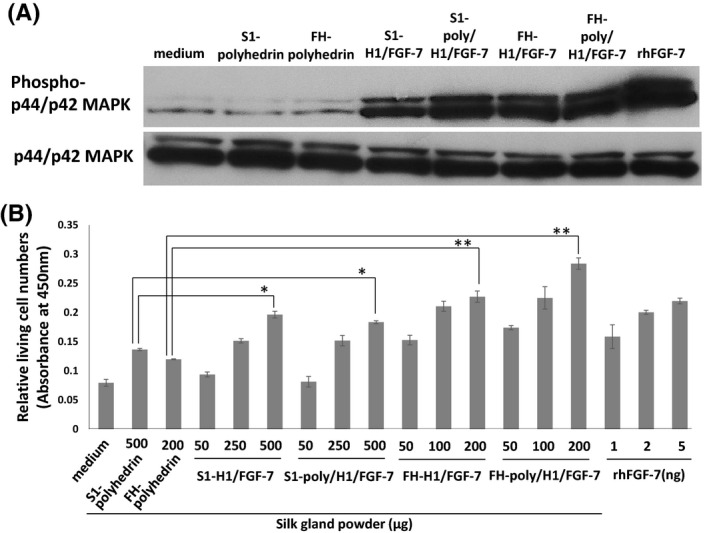
Proliferation of NHEK induced by silk gland powders (SGPs) containing H1/FGF‐7‐polyhedra. A, Phosphorylation of p44/42 mitogen‐activated protein kinase (MAPK) in cultured NHEKs. NHEKs were supplement‐starved overnight and left untreated as a negative control (no SGPs; lane 1) or treated with 10 mg of MSGPs from S1‐polyhedrin (lane 2), S1‐H1/FGF‐7 (lane 4) or S1‐poly/H1/FGF‐7 larvae (lane 5), or 10 mg of PSGPs from FH‐polyhedrin (lane 3), FH‐H1/FGF‐7 (lane 6), or FH‐poly/H1/FGF‐7 larvae (lane 7) for 1 h. NHEKs were also treated with 100 ng/ml rhFGF‐7 (lane 8) as a positive control. The NHEK lysates were examined using immunoblotting with antibodies against either p44/p42 MAPK or phosphorylated p44/p42 MAPK. B, Keratinocyte proliferation. Relative numbers of living cells were determined using a WST‐8 assay. NHEKs were cultured in media containing rhFGF‐7 (1 ng, 2 ng or 5 ng) or cultured with cell culture inserts with middle silk gland powders from S1‐polyhedrin (500 µg), S1‐H1/FGF‐7 (50, 250, or 500 µg) or S1‐poly/H1/FGF‐7 larvae (50, 250, or 500 µg), or posterior silk gland powders from FH‐polyhedrin (200 µg), FH‐H1/FGF‐7 (50, 100, or 200 µg) or FH‐poly/H1/FGF‐7 larvae (50, 100, or 200 µg). After incubation for 72 h, cell numbers were analyzed using a WST‐8 assay. Absorbance at 450 nm was determined using a microplate reader. Data are the mean ±SD of triplicate assays. *p < 0.05, **p < 0.01

FGF‐7 stimulation induces intracellular signaling events such as phosphorylation of receptor‐mediated p44/p42 mitogen‐activated protein kinase (MAPK).^13^ To assess the biological activity of H1/FGF‐7, MSGPs or PSGPs were added to the medium of supplement‐starved NHEKs through a cell culture insert filter and incubated for 1 h before immunoblotting of cell lysates with a phospho‐p44/p42 MAPK‐specific antibody (Figure 5A). Phosphorylation of NHEK p44/p42 MAPK was induced using MSGPs from S1‐H1/FGF‐7 (lane 4) and S1‐poly/H1/FGF‐7 (lane 5), and PSGPs from FH‐H1/FGF‐7 (lane 6) and FH‐poly/H1/FGF‐7 larvae (lane 7) as well as by rhFGF‐7 as a positive control (lane 8). No increase in phosphorylation was observed with no SGPs (lane 1), or with MSGPs from S1‐polyhedrin (lane 2) or PSGPs from FH‐polyhedrin larvae (lane 3; Figure 5A). There was a ~ 2 mm distance between cells and SGPs in cell culture insert filters. These results indicate that biologically active H1/FGF‐7 released from the SGPs could diffuse into the medium and pass through the filter to stimulate p44/p42 MAPK phosphorylation to reach levels that were comparable to those seen for rhFGF‐7.

After the culture of supplement‐starved NHEKs with SGPs for 72 h, cell proliferation was determined with a WST‐8 assay to detect the levels of intracellular dehydrogenase. The NHEKs were cultured with rhFGF‐7 as a positive control, or with no SGPs, MSGPs from S1‐polyhedrin or PSGPs from FH‐polyhedrin larvae as negative controls. The SGPs from S1‐H1/FGF‐7, S1‐poly/H1/FGF‐7, FH‐H1/FGF‐7, and FH‐poly/H1/FGF‐7 larvae all dose‐dependently induced NHEK proliferation to levels comparable to those seen for the positive control rhFGF‐7 (Figure 5B). There was a significant increase in the relative number of living cells in cultures treated with 500 µg MSGPs incorporating H1/FGF‐7 (S1‐H1/FGF‐7: ~1.4‐fold increase, *p* < 0.05; S1‐poly/H1/FGF‐7: ~1.3‐fold increase, *p* < 0.05) or 200 µg of PSGPs (FH‐H1/FGF‐7: ~1.9‐fold increase, *p* < 0.01; FH‐poly/H1/FGF‐7: ~2.4‐fold increase, *p* < 0.01) over that seen for cells treated with the same amount of SGPs without H1/FGF‐7 (Figure 5B). However, the relative amount of cell proliferation induced by MSGPs from S1‐H1/FGF‐7 and S1‐poly/H1/FGF‐7 larvae was lower than that for PSGPs from FH‐H1/FGF‐7 and FH‐poly/H1/FGF‐7 larvae, even though higher amounts of MSGPs were added (50, 250, and 500 µg vs. 50, 100, and 200 µg). The activities inducing the cell proliferation of the PSGPs from FH‐H1/FGF‐7 and FH‐poly/H1/FGF‐7 larvae were nearly fivefold higher than that of same amount of MSGPs from S1‐H1/FGF‐7 and S1‐poly/H1/FGF‐7 larvae. The PSGPs from FH‐poly/H1/FGF‐7 larvae also induced more cell proliferation than did FH‐H1/FGF‐7 (Figure 5B). Overall, these results demonstrate that H1/FGF‐7 released from both MSGPs and PSGPs is biologically active and can stimulate keratinocyte proliferation.

### 4.4 Construction of 3D cultures of keratinocytes with posterior silk gland powders incorporating H1/FGF‐7‐polyhedra

**FIGURE 6:**
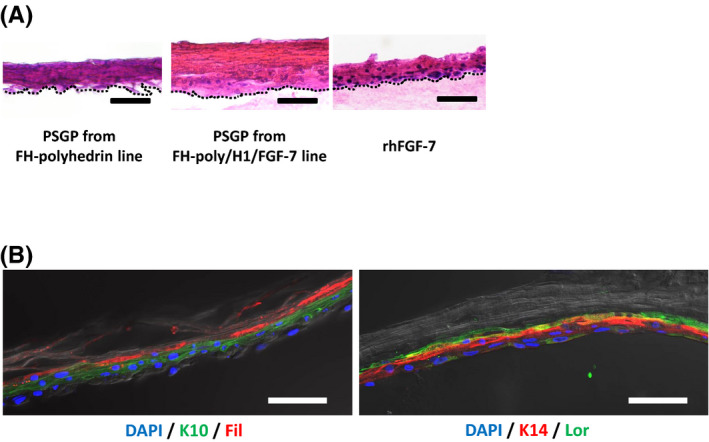
3D keratinocyte cultures. A, Hematoxylin and eosin (HE) staining in a 3D culture of keratinocytes. NHEKs were cultured on a collagen gel containing 800 µg posterior silk gland powders (PSGPs) from FH‐polyhedrin or FH‐poly/H1/FGF‐7 larvae in the basal medium for 2 days and in a differentiation medium (basal medium containing 1.2 mM Ca^2+^) for 14 days. The NHEKs were cultured in the same way on collagen with 100 ng/ml rhFGF‐7 in basal and differentiation media as a control. After cultivation for 14 days in differentiation media, 3D cultures were sectioned, subjected to HE staining and observed under a microscope. Scale bar, 50 µm. B, Immunohistochemical staining of keratinocyte 3D cultures. Immunohistochemical staining of keratin 10 (K10), keratin 14 (K14), Loricrin (Lor), and Filaggrin (Fil) in 3D cultures of keratinocytes incubated with PSGPs from FH‐poly/H1/FGF‐7 larvae. Nuclei were stained with 4′,6‐diamidino‐2‐phenylindole. Scale bar, 50 µm

Three‐dimensional NHEK cultures can be used as a model of human epidermis to substitute for in vivo tests involving animals, which successfully assess skin irritations caused by the acute toxicity,^31^, ^32^ allergenicity,^33^ and inflammation^34^ of test compounds. We used PSGPs with H1/FGF‐7 polyhedra to construct a 3D epidermis model to examine the gradual release of H1/FGF‐7 from these polyhedra. The NHEKs were cultured on type 1 collagen gels containing PSGPs from FH‐polyhedrin or FH‐poly/H1/FGF‐7 larvae in a basal medium without added rhFGF‐7. After replacing the basal medium with a differentiation medium containing Ca^2+^, air‐liquid‐interface cultivation was continued for 14 days. The NHEKs were also cultured on gels with basal media and differentiation media with rhFGF‐7 and without added PSGPs. The differentiation media were replaced twice weekly. NHEK differentiation was induced at the air‐liquid interface, and the enucleation of keratinocytes was verified by HE staining (Figure 6A). The 3D cultures with added PSGPs of FH‐polyhedrin larvae and rhFGF‐7 did not induce sufficient proliferation and adequate differentiation based on negative HE staining and a lack of an enucleated cell layer in the upper parts of the cell culture. However, microscopic observation of the 3D culture of HE‐stained NHEK treated with PSGPs from FH‐poly/H1/FGF‐7 larvae did reveal sufficient cell proliferation and a well‐developed upper layer of enucleated cells corresponding to epidermal morphogenesis (Figure 6A).

NHEK 3D cultures with PSGPs from FH‐poly/H1/FGF‐7 larvae were further examined using immunofluorescence (Figure 6B). Cells in the 3D culture were stained with antibodies for keratin 14, which is a stratum basal layer marker, keratin 10, which is a stratum spinosum marker, and loricrin and filaggrin, which are stratum corneum‐specific markers. Keratin 10 and keratin 14 staining demonstrated that keratinocyte differentiation and localization were induced during cultivation. Similarly, the loricrin‐ and filaggrin‐positive layer exhibited a well‐formed stratum corneum in 3D cultures of NHEK treated with FH‐poly/H1/FGF‐7 larvae PSGPs (Figure 6B), demonstrating that H1/FGF‐7 released from PSGPs could induce epidermal morphogenesis with the expected expression of epidermal markers.

## 5 DISCUSSION

In this study, we report that the generation of transgenic silkworms with silk gland cells containing CPV polyhedra that encapsulate H1/FGF‐7. H1/FGF‐7 and polyhedrin genes were controlled by either the *FH* or *S1* promoter, which are both upregulated upon ecdysteroid secretion, which occurs during the late stage of the 5th instar. The amount of H1/FGF‐7‐polyhedra in the silk glands is predicted to be maximized during the spinning stage after a gut purge, as was previously observed for the production of FGF‐2.^7^ Thus, biologically active H1/FGF‐7 would be present in polyhedra in SGP during the spinning stage of larval development.

Immunofluorescence detected with an FGF‐7 antibody showed specific H1/FGF‐7 signals near the polyhedra surface (Figure 3B). FGF‐7 has a very short half‐life of biological activity due to low protein stability.^13^, ^14^ However, the assays of NHEK proliferation that were performed for this study demonstrated that long‐term, stable H1/FGF‐7 activity was maintained in PSGPs (Figure 5). Moreover, SGPs with H1/FGF‐7‐polyhedra induced a higher amount of NHEK proliferation than SGPs containing only H1/FGF‐7 without polyhedra (Figure 5), demonstrating that encapsulation of H1/FGF‐7 in polyhedra in transgenic PSGs could maintain stable bioactivity of H1/FGF‐7 in the silkworm body or SGPs. It is still unclear whether the addition of an H1‐tag to the N‐terminus of FGF‐7 affects its ability to induce keratinocyte proliferation. However, we did not find a remarkable loss in the bioactivity of H1/FGF‐7 through the addition of H1‐tag in this and a previous study.^16^

Research has shown that degummed fibroin can preserve cell growth factor activity even after processing solubilized fibroin into recrystalized sponges or films.^5^, ^6^ In addition, crystalized fibroin, which forms a layer of fibroin in silkworm cocoons, can protect the bioactivity of recombinant proteins expressed in PSGs, or those secreted into the PSG lumen and subsequently embedded in fibroin crystals during spinning.^1^, ^2^, ^3^ In this study, H1/FGF‐7 was not secreted into the fibroin layer of the cocoon. Instead, it was likely embedded in fibroin crystals formed during freeze‐drying.^38^ H1/FGF‐7 activity was stably maintained during physical processing of the fibroin such that PSGs with free H1/FGF‐7 could still induce NHEK proliferation (Figure 5). From this perspective, the activity of cytokines contained in PSGs withstands physical processing. Weak activities were shown in the media with MSGPs from S1‐polyhedrin or PSGPs from FH‐polyhedrin larvae after the 3 day incubation, as with the previous reports that fibroin^39^ and sericin^40^ of silkworm SGPs have slight activities to stimulate mammalian cell proliferation.

We also found that MSGPs incorporating H1/FGF‐7‐polyhedra induced NHEK proliferation, although to a lesser extent than that observed for PSGPs. One possible explanation for this weaker activity is that silk proteins in liquid form in PSGs are carried into and accumulate in MSGs such that the relative concentration of H1/FGF‐7 in MSGPs would be lower than that in PSGPs. However, MSGs can be processed into fibrous materials, such as silk guts, because of the high amount of liquid fibroin they contain. For effective use of MSG fibrous materials to control cell proliferation, systems to express foreign proteins in MSGs could be improved through various modifications, such as enhanced promoter activity, which is achieved by the introduction of a baculovirus *hr3* sequence in the region upstream of the promoter.^1^, ^3^

We also showed that PSGPs with H1/FGF‐7‐polyhedra continued to release H1/FGF‐7 for at least 14 days, as with H1/FGF‐7‐polyhedra (Figure 4). Posterior silk gland materials that incorporate polyhedra that encapsulate cytokines could thus serve as effective cytokine carriers for tissue engineering applications and could be used as a platform to continuously release diverse cytokines. In addition, PSGPs incorporating H1/FGF‐7‐polyhedra can be used in the construction of a human epidermis model that requires sustainable supplies of cell growth factors to achieve long‐term cultivation (Figure 6). Typically, epidermal cultures require fresh media that are supplemented with fresh cytokines every 2‐3 days.^35^, ^36^, ^37^ NHEK 3D cultures that maintain an air‐liquid interface over the long term are challenging to manipulate, and the replacement of medium can inhibit the appropriate formation of the epidermis. Here we revealed that the introduction of H1/FGF‐7‐polyhedra‐incorporating PSGPs effectively induced keratinocyte proliferation and differentiation into a stratified epidermis that expressed the expected epidermal differentiation protein markers (Figure 6A, B). As described, the medium of the 3D culture was changed every 3‐4 days (twice weekly), but stratified epidermis did not form with the addition of only rhFGF‐7 (Figure 6A), suggesting the need for more frequent replacement of medium supplemented with rhFGF‐7 than was previously recommended.^35^, ^36^, ^37^ We showed that, even with infrequent medium replacement, PSGP‐containing polyhedra that incorporate H1/FGF‐7 induced keratinocyte proliferation and differentiation into the stratified epidermis. Thus, polyhedra incorporating H1/FGF‐7 could be a useful tool for generating and maintaining 3D NHEK cultures for use as in vitro epidermal models.

Here we used H1/FGF‐7‐polyhedra‐incorporating PSGPs to control the proliferation and differentiation of NHEKs, although methods involving lysis, crushing, and sonication of SGPs have recently been developed to prepare polyhedra from MSGPs of transgenic silkworms.^4^ Using these steps to remove the PSG component enables the use of prepared polyhedra from individual silkworms as an effective protein production host for tissue engineering. Cytokine‐encapsulated polyhedra have typically been isolated from recombinant baculovirus‐infected insect cultured cells, although such culture systems do raise concerns about the presence of amphixenotic pathogens that could contaminate animal serum added to cell culture media. In addition, baculovirus shows some immunostimulatory activity toward mammalian cells.^41^ The foreign protein expression system we used in transgenic silkworms does not involve viral vectors or animal serum for in vitro cell culture. Therefore, it is useful for generating artificial materials that mimic the extracellular matrices or cell scaffolds that locally release cell growth factors. Such materials would be valuable for grafts and other applications. The sericulture industry has effective systems for the large‐scale production of SGPs that can be used to produce not only in vitro cell culture models but also artificial materials for clinical use.^12^

In conclusion, we describe the genetic engineering of a silkworm line for the production of posterior silk gland materials that incorporate FGF‐7 protein microcrystals that protect and release FGF‐7 activity. These silk gland materials have the potential to control keratinocyte proliferation and differentiation for the generation of human epidermis models for in vitro applications. The results of this study will be useful for the development of silk gland materials for cell engineering purposes, both in vivo and in vitro.

## AUTHOR CONTRIBUTIONS

E. Kotani supervised and directed the work; E. Kotani, R. Matura, and K. Takaki designed the research; R. Maruta, Y. Yamaji, and K. Takaki performed the research; H. Sezutsu and H. Mori contributed new analytic tools. R. Maruta and E. Kotani interpreted the data and wrote the manuscript.

## Supporting information

Supplementary Material
