# Supplementary material for: Effects of transgenic silk materials that incorporate FGF‐7 protein microcrystals on the proliferation and differentiation of human keratinocytes
Source: FASEB Bioadv. 2020 Oct 20;2(12):734–44. doi: 10.1096/fba.2020-00078 (PMC7734426; doi:10.1096/fba.2020-00078)
Supplement: Supplementary file 1 — Supplementary Material [file FBA2-2-734-s001.pdf]

SUPPLEMENTAL RESULTS

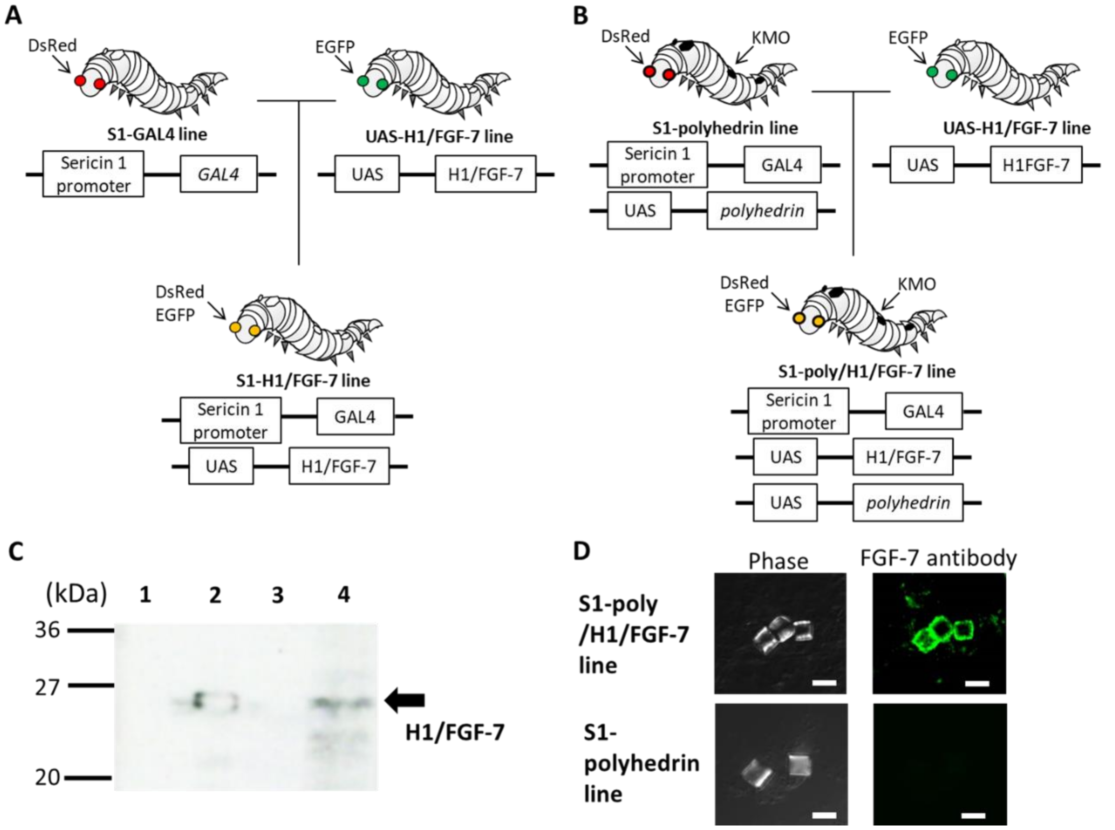

Supplemental Figure S1. Generation of transgenic silkworms expressing H1/FGF-7 in the MSG. *A*) Generation of the S1-H1/FGF-7 line. The S1-GAL4 line (30) was mated with the UAS-H1/FGF-7 line to generate the S1-H1/FGF-7 line with EGFP and DsRed expression in the eyes. *B*) Generation of the S1-poly/H1/FGF-7 line. The S1-polyhedrin line was mated with the UAS-H1/FGF-7 line to generate the S1-poly/H1/FGF-7 line with EGFP and DsRed expression in the eyes and KMO-specific skin (7). *C*) Immunoblotting analysis of H1/FGF-7 expression in MSGs with 12.5% SDS-PAGE and anti-FGF-7 antibodies. Lane 1 and 2: Protein from 50,000 cubes of empty polyhedra (negative control) and FGF-7-polyhedra (positive control) produced in baculovirus-infected Sf21 cells, respectively; Lanes 3 and 4: Immunoprecipitated protein from 50 mg MSGs from *w1-pnd* and S1-H1/FGF-7 larvae, respectively. Protein size markers are indicated. *D*) Confocal immunofluorescence microscopy analysis of H1/FGF-7 on polyhedra. Polyhedra collected from S1-poly/H1/FGF-7 or S1-polyhedrin larvae MSGs were fixed on a glass-based dish and examined by an immunofluorescence with an anti-FGF-7 antibody. Scale bars, 10 µm.

Supplemental Table S1. Inverse PCR of the UAS-H1/FGF-7 transgene border sequence in the *w1-pnd* genome and sequence analysis with the Kaikoblast database (http://kaikoblast.dna.affrc.go.jp). The chromosome number and clone name are listed for the border sequence. The TTAA consensus sequence appearing at the border of the *piggyback*-driven transgene is underlined.

Silkworm line	Sequence at the transgene border	Chromosome no. (clone name)
UAS-H1/FGF-7	CAAGAATATTAA(transgene)TTAATAAACTACCA	Chromosome 8 (Bm_scaf51)
